# Epitope Mapping and Fine Specificity of Human T and B Cell Responses for Novel Candidate Blood-Stage Malaria Vaccine P27A

**DOI:** 10.3389/fimmu.2020.00412

**Published:** 2020-03-10

**Authors:** Kristina M. Geiger, Daniel Guignard, Che Yang, Jean-Pierre Bikorimana, Bruno E. Correia, Sophie Houard, Catherine Mkindi, Claudia A. Daubenberger, François Spertini, Giampietro Corradin, Régine Audran

**Affiliations:** ^1^Biochemistry Department, University of Lausanne, Epalinges, Switzerland; ^2^Department of Infectious Diseases and Immunity, University of California, Berkeley, Berkeley, CA, United States; ^3^Institute of Bioengineering, École Polytechnique Fédérale de Lausanne, Lausanne, Switzerland; ^4^Swiss Institute of Bioinformatics, Lausanne, Switzerland; ^5^Division of Immunology and Allergy, Centre Hospitalier Universitaire Vaudois, Lausanne, Switzerland; ^6^European Vaccine Initiative, Heidelberg, Germany; ^7^Swiss Tropical and Public Health Institute, Basel, Switzerland; ^8^University of Basel, Basel, Switzerland; ^9^Department of Intervention and Clinical Trials, Ifakara Health Institute, Bagamoyo, Tanzania

**Keywords:** malaria, *Plasmodium falciparum*, vaccine, P27A, clinical trial, immune response, adjuvant, populations

## Abstract

P27A is a novel synthetic malaria vaccine candidate derived from the blood stage *Plasmodium falciparum* protein Trophozoite Exported Protein 1 (TEX1/PFF0165c). In phase 1a/1b clinical trials in malaria unexposed adults in Switzerland and in malaria pre-exposed adults in Tanzania, P27A formulated with Alhydrogel and GLA-SE adjuvants induced antigen-specific antibodies and T-cell activity. The GLA-SE adjuvant induced significantly stronger humoral responses than the Alhydrogel adjuvant. Groups of pre-exposed and unexposed subjects received identical vaccine formulations, which supported the comparison of the cellular and humoral response to P27A in terms of fine specificity and affinity for populations and adjuvants. Globally, fine specificity of the T and B cell responses exhibited preferred recognized sequences and did not highlight major differences between adjuvants or populations. Affinity of anti-P27A antibodies was around 10^−8^ M in all groups. Pre-exposed volunteers presented anti-P27A with higher affinity than unexposed volunteers. Increasing the dose of GLA-SE from 2.5 to 5 μg in pre-exposed volunteers improved anti-P27A affinity and decreased the number of recognized epitopes. These results indicate a higher maturation of the humoral response in pre-exposed volunteers, particularly when immunized with P27A formulated with 5 μg GLA-SE.

## Introduction

In 2018, malaria affected 213 million people and caused 405,000 deaths worldwide. Sub-Saharan Africa accounts for nearly 93.4% of total malaria cases. Children under 5 years of age are most susceptible to malaria and account for 67% of all malaria deaths (1). *Plasmodium falciparum* (*Pf*) causes the most serious forms of malaria. Though there are treatments and promising vaccine candidates for this disease, a highly efficacious vaccine is not currently available (2, 3). Sequencing of the *Pf* genome provided over 5,300 potential gene and protein targets for drug and vaccine development (4). Using a genome-wide approach, new protein fragments have been identified with defined structural features that are potential targets for antibodies that inhibit parasite growth (5). One of these fragments, P27A, consists of 104 amino acid residues (aa) and is part of *Pf* Trophozoite Exported Protein 1, TEX1/PFF0165c (5–7). This novel fragment is particularly interesting because its sequence is highly conserved with a single mutation E/G at position 292 (5, 7). The P27A amino acid sequence indicates that the fragment assumes an intrinsically unstructured conformation, which was further established by circular dichroism studies (5). Unstructured antigens/fragments that consist of over 50 amino acid residues and exhibit biological activity were first reported by Wright and Dyson (8), followed by reports of other fragments specifically relevant to the *plasmodia* parasite and other organisms by Feng et al. and Corradin et al. (9–11). Purified human antibodies with P27A-specific affinity inhibit parasite activity *in vitro*, and the anti-P27A response is associated with protection (5, 7). Peptide vaccines induce a direct, strong immune response, and are cost-effective and easy to produce and store (12–14). Due to the promising properties of P27A as an anti-malaria vaccine candidate, phase 1a and 1b clinical trials were performed in malaria unexposed subjects from Switzerland (CH) and in malaria pre-exposed healthy adults from Tanzania (TZ) to evaluate its safety and immunogenic properties in combination with adjuvants Alhydrogel or glucopyranosyl lipid A in a stable emulsion (GLA-SE) (15). The vaccine was safe and induced a strong humoral response, particularly in combination with GLA-SE, and was active in inhibiting *in vitro* parasite growth (15). In this report, we characterized the anti-P27A cellular and humoral responses generated in vaccinated subjects, particularly the fine specificity and IgG affinities. Groups of pre-exposed and unexposed subjects received identical vaccine formulations, which supported comparisons of populations and adjuvants.

## Materials and Methods

### Peptides for *in vitro* Use

Ten 20-mer synthetic peptide fragments overlapping on 10 amino acids (P1–P10) were synthesized using the solid-phase Fmoc strategy (Table 1) (6, 12). Peptides were purified by reverse-phase high-pressure liquid chromatography and analyzed by mass spectrometry (6). P27A GLP and GMP preparations (Almac, Craigavon, UK) were used throughout the study. For cell stimulation, lyophilized peptides were resuspended in apyrogenic sterile water and filtered through a 0.22 μm filter.

**Table 1:** Sequences of P27A and overlapping 20-mers peptides.

**Peptide**	**PFF0165c**	**Sequence**
P27A-LSP	223–326	HNNNEKNISY//QEEKENMLNNKKRS
P1	223–242	HNNNEKNISYDKNLVKQEND
P2	233–252	DKNLVKQENDNKDEARGNDN
P3	243–262	NKDEARGNDNMCGNYDIHNE
P4	253–272	MCGNYDIHNERGEMLDKGKS
P5	263–282	RGEMLDKGKSYSGDEKINTS
P6	273–292	YSGDEKINTSDNAKSCSGDE
P7	283–312	DNAKSCSGDEKVITSDNGKS
P8	293–322	KVITSDNGKSYDYVKNESEE
P9	303–322	YDYVKNESEEQEEKENMLNN
P10	313–332	QEEKENMLNNKKRSLECNPN

*Underlined amino acids in P10 are not present in P27A-LSP sequence*.

### Human Samples

P27A formulated in Alum or in GLA-SE was studied in parallel in a phase 1a clinical trial in Lausanne, Switzerland (CH) and in a 1b clinical trial in Bagamoyo, Tanzania (TZ) (15). Trials were registered in clinicaltrials.gov (NCT01949909) and www.pactr.org (PACTR201310000683408). As described in Figure S1, two groups from CH and three groups from TZ, each consisting of eight healthy individuals, received three intramuscular (i.m.) injections at days 0, 28, and 56. Volunteers of groups CH-Alum/50 and TZ-Alum/50 received 50 μg P27A and 0.85 mg Alhydrogel adjuvant, per injection. In groups CH-GLA2.5/50, TZ-GLA2.5/50, and TZ-GLA5/50, they received 50 μg P27A and 2.5 μg or 5 μg GLA-SE. Sera, plasma and peripheral blood mononuclear cells (PBMC) were taken at day 0 (D0, pre-vaccination) and at day 84 (D84, 1 month after the third injection) using the same procedure at both sites. PBMC were separated from blood on a density gradient (Ficoll-Paque Plus) and were stored in liquid nitrogen until use. Positive controls were a pool of sera from eight exposed subjects from Burkina Faso, set arbitrarily to 152 AU/mL to P27A, and the plasma of a vaccinated volunteer with a high response (5,420 AU/mL to P27A). Negative control sera were pooled from 20 non-exposed individuals.

### ELISPOT Determination

After thawing, PBMC were plated in 96-round-bottom well plates at 2.5 × 10^5^ cells/well in 8% human AB serum in RPMI. Cells were stimulated separately in triplicates with each of the following: 50 μg/mL of P27A, 10 μg/mL of each 20-mer P27A peptide, a pool of the ten 20-mer peptides (10 μg/mL of each), 5 μg/mL of phytohaemagglutinin (PHA) and unstimulated as controls. For the stimulation with PHA, 40,000 to 75,000 cells were plated. After 20–24 h of culturing at 37°C 5% CO_2_, cells were transferred for a 15–20 h supplementary culturing on a nitrocellulose-lined microtiter plate coated with 100 μL of capture anti-hu IFN-γ (5 μg/mL) and blocked (BD™ Human IFN-gamma ELISPOT Set). At the end of culture, the plate was washed and incubated for 2 h at room temperature (RT) with 100 μL/well of biotinylated anti-hu IFN-γ antibody (2 μg/mL, BD). After washing, plate was incubated with 100 μL/well of streptavidin-HRP conjugate (BD) for 1 h at RT. After washing, 100 μL/well of 3-amino-9-ethyl-carbazole (AEC, BD^TM^ AEC substrate reagent set) was added for a 5–30 min incubation stopped with tap water. After plate drying, spots were counted using a computer assisted video image analyzer (EliSpot Robotic Systems with AID EliSpot Software Version 6.x, ELROBO6i, AID, D-Straßberg). Results were expressed as spot forming units (SFU)/million PBMC.

### Humoral Response

The immunoglobulin G (IgG) immune response against the synthetic P27A and overlapping 20-mers was measured using an enzyme-linked immunosorbent assay (ELISA) (5, 15). Microtiter plates (Nunc™ Maxisorp, Denmark) were coated at a concentration of 2 and 5 μg/mL, respectively, at RT for 1 h then blocked with PBS-3% milk for 1 h. Samples and controls were serially diluted in PBS-1.5% milk-0-05% Tween and added at 50 μL per well. Controls were present on each ELISA plate. Anti-hu IgG secondary antibodies conjugated to alkaline phosphatase (A-9544, Sigma, St. Louis, MO), diluted 1:1000 in PBS-1.5% milk-0-05% Tween, were added to each well. p-Nitrophenyl phosphate substrate tablets (Sigmafast™, Sigma, St. Louis, MO) were dissolved and added to each well. The optical density (OD) of each well was measured at 405 nm using an OpsysMR plate reader (Dynex Technologies). Results were expressed as AU/mL based on the standard curve obtained from the response of the positive control pool of sera to P27A.

### ELISA-Based Determination of Apparent Affinity (EC50) and Avidity

EC50 represents the concentration of soluble peptide inhibitor required to obtain 50% inhibition of IgG binding to coated peptide (16). The competitive ELISA protocol (17–19) was adapted and modified. 96-well microtiter plates (Nunc™ Maxisorp, Denmark) were coated with 50 μL of 2 μg/mL of P27A at RT for 1 h then blocked with PBS-3% milk for 1 h. Sera samples, at the dilution giving 50% of the maximal binding to coated P27A, were incubated 30 min at RT with 1:10 serial dilutions of competitor soluble P27A or 20-mer peptides starting with concentrations of either 200 μg/mL or 10^5^ M and without peptide as control (max). Then, 50 μL of each well was plated in duplicates onto the P27A coated plate, incubated at RT for 2 h and developed as described above. Percentage of inhibition was calculated as: 100-(OD_i_–OD_min_)/(OD_max_–OD_min_) × 100, OD_min_ representing signal in well without serum. EC50 and EC10 were calculated using the linear function: % inhibition = f [log (peptide concentration)]. Using the same method as for affinity, avidity was evaluated using 1:2 serial dilutions of the chaotropic reagent guanidine hydrochloride (GuHCl, from 6M to 0.1 M) as inhibitor and without GuHCl as control (max). Avidity was expressed as the concentration of GuHCl (M) required to obtain 50% inhibition of IgG binding to coated P27A.

### Affinity Purified Human Antibodies

P27A-sepharose conjugate was prepared with 16 mg of P27A mixed with 2.5 g of CNBr-Sepharose 4B (Amersham Bioscience AB, S-Uppsala) according to the manufacturer's protocol. Five to 7 mL of D84 plasma diluted 1:5 in PBS 0.5 M NaCl was mixed with the P27A-sepharose conjugate and stirred gently overnight at 4°C. After centrifugation, washing and transferring the beads into a syringe, bound antibodies were eluted with glycine (0.1 M, pH 2.5). The collected fractions were instantly neutralized with PBS (10x, pH 7.4), and their protein concentration was determined by the absorbance at 280 nm and anti-P27A IgG by ELISA. Fractions of interest were pooled. Total IgG from D0 plasma were purified by affinity chromatography on protein G using the manufacturer's protocol (GammaBind™ Plus Sepharose™, GE Healthcare, S-Uppsala).

### Surface Plasmon Resonance Measurements of Avidity in Human Sera

A Biacore 8K instrument (GE Healthcare, Glattbrugg, Switzerland) was used to determine the apparent dissociation constant (K_D_) of antigen-specific human polyclonal antibodies to P27A. Around 10 μg/mL of P27A in 10 mM sodium acetate, pH = 4.5 was immobilized on a CM5 chip to reach the final chip density of about 150 response units (RU). Affinity-purified antibodies from volunteers were 1:2 serially diluted from 1 μM down to 200 pM and injected at a flow rate of 10 μL/min. After each cycle of injection, the surface chip was regenerated by flushing it with 3 M MgCl for two consecutive 30-second intervals. Association (k_on_), dissociation (k_off_) and equilibrium (K_D_) constants were determined using 1:1 Langmuir binding kinetic fits provided by the suite of analysis software from Biacore 8K (GE Healthcare #29310604).

### Statistical Analysis

The data were analyzed using GraphPad Prism 7.03. Wilcoxon and Mann Whitney non-parametric tests were performed to compare intra- and inter- group values, respectively. Spearman and Pearson correlations between parameters were also performed. A two-way ANOVA test was used to compare antibody titers of each adjuvant group against each peptide. The significance level used for each test was α = 0.05.

## Results

### T-Cell Response and Fine-Specificity

**Figure 1:**
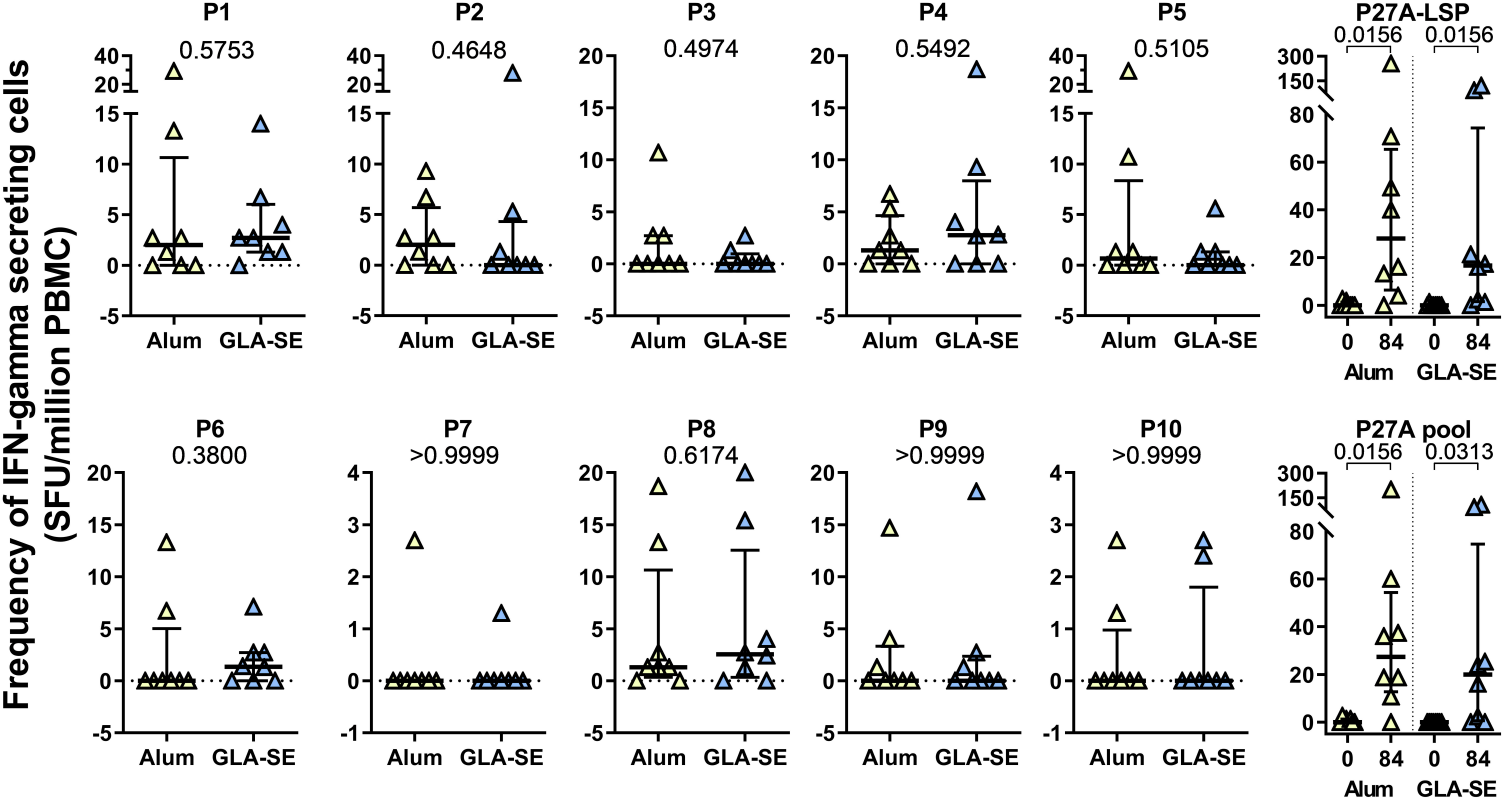
Cellular responses to P27A and overlapping 20-mers at D84 in Phase 1a. PBMC from unexposed volunteers (CH-Alum/50 (*n* = 8) and CH-GLA2.5/50) (*n* = 8) at D84 were stimulated with P27A-LSP or the overlapping 20-mer peptides P1 to P10, individually or as a pool. The frequency of IFN-γ secreting cells was revealed by ELISPOT. Results are expressed as SFU/ million PBMC. Comparisons between groups were done using Mann-Whitney test; *p-*values are indicated. Responses at D0 and D84 to P27A-LSP and the pool of peptides are shown with Wilcoxon test *p*-values.

**Figure 2:**
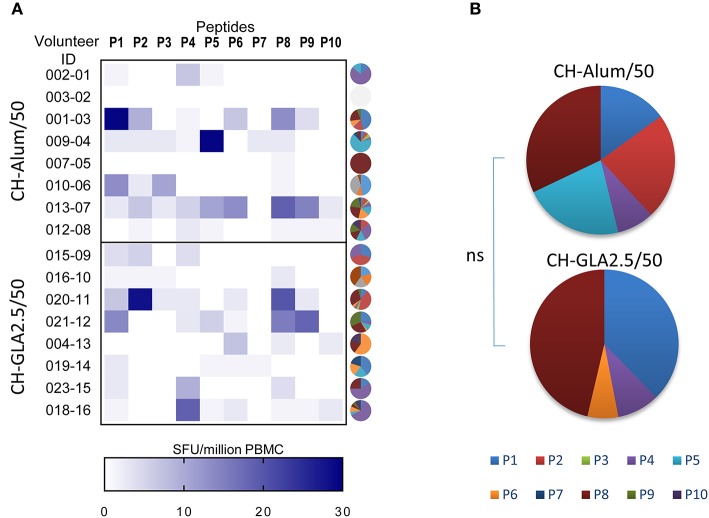
Repartition of the cellular recognition along the sequence of P27A at D84 in Phase 1a. PBMC from unexposed volunteers [CH-Alum/50 (*n* = 8) and CH-GLA2.5/50 (*n* = 8)] at D84 were individually stimulated with overlapping 20-mer peptides P1 to P10, and the frequency of IFN-γ secreting cells revealed by ELISPOT. **(A)** Heat map indicates the individual responses to each 20-mer peptide,Y-axis represents the volunteer ID and the X-axis represents the frequency of IFN-γ secreting cells as SFU/million PBMC, and individual pies illustrate the repartition of responses to the 20-mers as % of the total response. **(B)** Pie graphs show the median value per vaccination group of the percentage of IFN-γ secreting cells to each 20-mer peptide.

The effect of adjuvants on the T-cell response was evaluated in unexposed volunteers. The IFN-γ response to each 20-mer peptide, individual or pooled, or to the full length P27A (P27A-LSP) was determined by ELISPOT at D0 and D84 (Figure 1). Volunteers from the groups CH-Alum/50 and CH-GLA2.5/50 exhibited a significant IFN-γ response to the pool of 20-mer peptides and P27A-LSP. The two adjuvant groups of unexposed volunteers had similar IFN-γ responses. The average response to the pool of 20-mer peptides was 27.35 [0; 200] SFU/million PBMC for the CH-Alum/50 group and 20 [0; 106.7] SFU/million PBMC for the CH-GLA2.5/50 group; Wilcoxon *p-*values were 0.0156 and 0.0313, respectively. The average response to P27A-LSP was 28 [0; 256] SFU/million PBMC for the CH-Alum/50 group and 16.65 [0; 120.4] SFU/million PBMC for the CH-GLA2.5/50 group; Wilcoxon *p-*values were 0.0156 and 0.0156, respectively. By comparison, the IFN-γ responses to individual 20-mer peptides was weaker, but the sum of the individual responses was about equal to that of the 20-mer peptide pool and P27A-LSP. No comparative difference in intensity was observed between the adjuvant groups. The distribution of the IFN-γ responses along the sequence varies widely between volunteers (Figure 2A). However, the resulting responses per group focused on 4–5 peptides, principally P1, P2, P5, and P8 with Alum and on P1 and P8 with GLA-SE (Figure 2B).

### Antibody Response and Fine-Specificity

**Figure 3:**
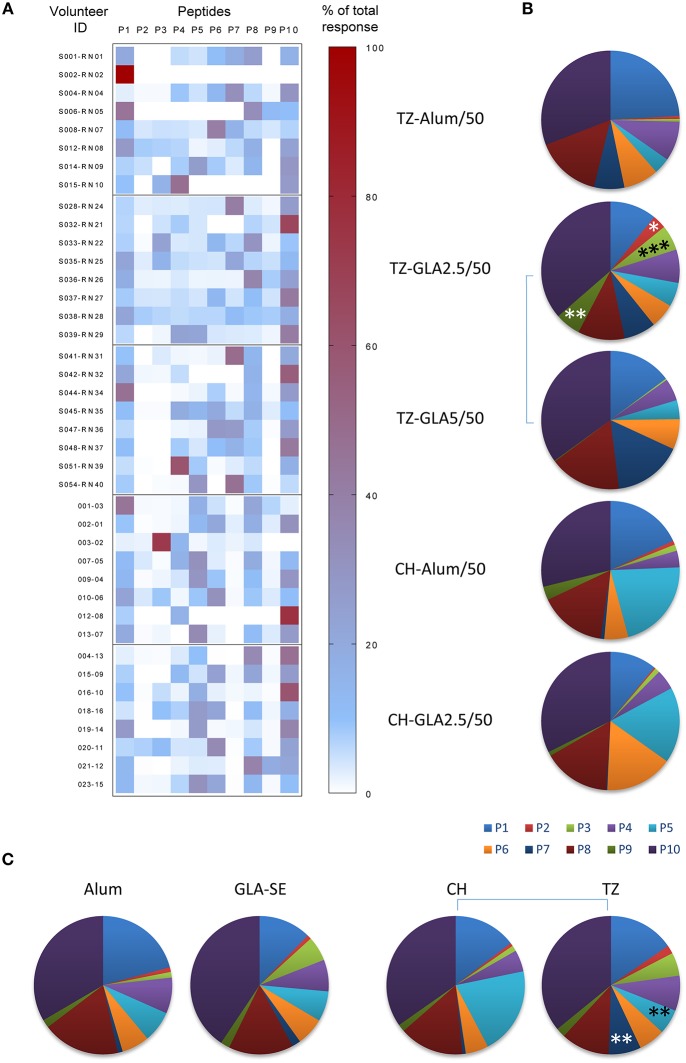
Repartition of the fine specificity of the humoral response along the sequence of P27A at D84 in Phase 1a and 1b. The fine specificity of the humoral response was determined by ELISA using sera from unexposed volunteers (CH-Alum/50 and CH-GLA2.5/50) and exposed volunteers (TZ-Alum/50, TZ-GLA2.5/50, and TZ-GLA5/50) against individual 20-mer peptides. **(A)** Heat map indicates the individual repartition of antibody responses to each 20-mer peptide, P1 to P10, over the sum of the responses, for each vaccination group. Right Y-axis represents the volunteer ID and the left Y-axis denotes the percentage value. **(B)** Pie graphs show the median proportion of humoral response to each 20-mer peptide for each vaccination group. **(C)** Pie graphs represent the median repartition of humoral responses of all volunteers grouped either by adjuvant type (Alum or GLA-SE) or by site (CH or TZ). Comparisons per peptide were done using Mann-Whitney test; *p < 0.05; **p < 0.01; ***p<0.001.

The fine specificity of the humoral response was determined by ELISA using individual 20-mer peptides. We previously reported that GLA-SE formulation induced higher anti-P27A titers than Alum, and that pre-exposed subjects exhibited lower humoral responses as compared to unexposed subjects. Furthermore, an increase in the dose of GLA-SE in the vaccine formulation was associated with an increase in antibody titers (5, 15). We observed that the antibody responses directed to the 20-mer peptides had the same intensity profile as anti-P27A. To characterize the fine specificity and compare responses between groups, we analyzed the proportion of responses to the 20-mers peptides. Figure 3A shows the percentage of recognition for each 20-mer peptide, and Figure 3B indicates the proportions of the responses to each peptide in the different groups. Comparisons among the two adjuvants used and the volunteers at the two sites are shown in Figure 3C. The most striking observations found are: (1) similarity of low or no responses to peptides P2, P3, and P9 in all groups (Figure 3A); (2) P10 is most frequently recognized followed by P1 and P8; (3) comparison of the TZ-GLA-SE2.5/50 and TZ-GLA-SE5/50 pie graphs shows that an increase in GLA-SE from 2.5 to 5 μg induced a significant reduction in the number of recognized peptides (*p* < 0.0001 in Kruskal Wallis, Figures 3B,C); and (4) globally, both formulations induced a similar pattern of humoral responses in the two populations, except to peptides P5 and P7. Recognition frequency of P5 was significantly lower in the pre-exposed group than in the unexposed group (*p* = 0.0098), while on the contrary, recognition frequency of P7 was significantly higher in the pre-exposed group than in the unexposed group (*p* = 0.0092), regardless of the adjuvant formulation (Figure 3C).

### Antibody Affinities and Avidities to P27A

**Figure 4:**
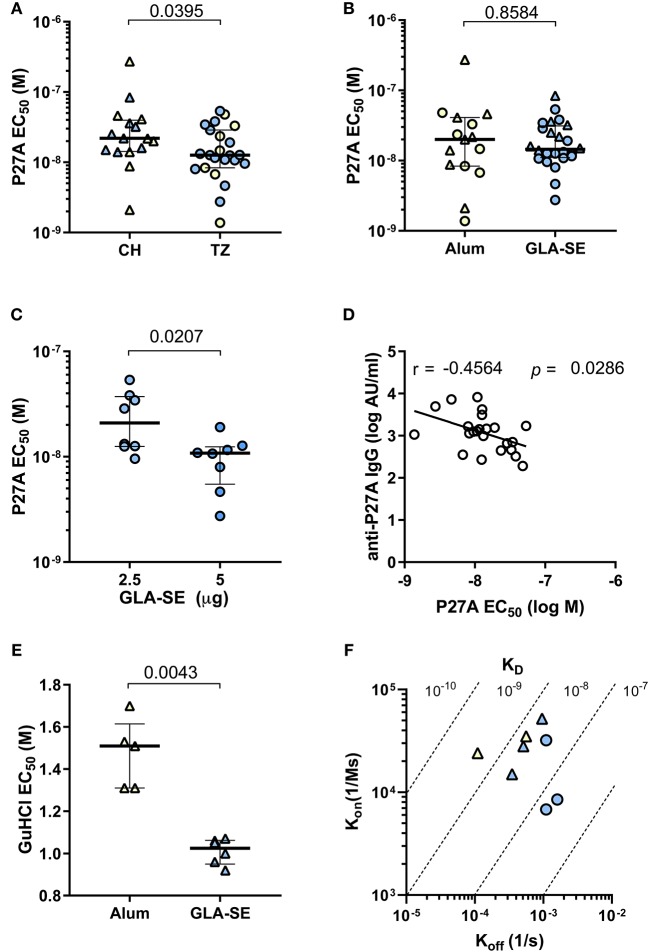
Relative affinity and avidity of specific IgG to P27A at Day 84 in Phase 1a and 1b. Strength of the binding of specific IgG to P27A was evaluated at day 84 for all volunteers (CH and TZ) by competition ELISA using soluble P27A as competitor **(A–C)** or guanidium chloride GuHCl **(E)**. Results are expressed as EC_50_. **(A)** Comparison of relative antibody affinity for P27A in pre-exposed and unexposed subjects, from TZ (3 groups, *n* = 23) and CH (2 groups, *n* = 16), respectively. **(B)** Comparison of relative antibody affinity for P27A in each adjuvant group, Alum (*n* = 16) and GLA-SE (*n* = 23). **(C)** Comparison of relative antibody affinity for P27A in each TZ-GLA-SE dose group, 2.5 or 5 ug. **(D)** Correlation between anti-P27A IgG titer (log AU/mL) and affinity (log M) in pre-exposed TZ subjects, *n* = 23. Pearson *r* and *p*-values are indicated. **(E)** Comparison of antibody avidity for P27A in unexposed volunteers grouped by adjuvant type, Alum (*n* = 5) and GLA-SE (*n* = 5). **(F)** Antibody avidity for P27A in eight samples from TZ-GLA2.5/50 (*n* = 3, blue circles), CH-GLA2.5/50 (*n* = 3, blue triangles), and CH-Alum/50 (*n* = 2, yellow triangles). Antibody avidity was determined by SPR with kinetic constants K_on_ (association) and K_off_ (dissociation) and equilibrium K_D_ = K_off_/K_on_. **(A–C,E)** Lines indicate medians and quartiles, *p*-values of Mann-Whitney tests are indicated. TZ subjects, circles; CH subjects, triangles; Alum, yellow symbols; GLA-SE, blue symbols.

We performed a competitive ELISA to evaluate the functional antibody affinity for soluble P27A of each volunteer after the third vaccination (Day 84). We observed a 2-log difference within individual EC_50_ (Figure 4). Figure 4A shows that the overall affinity for soluble P27A was significantly higher in the pre-exposed group than in the unexposed group (median EC_50_ of 1.26 × 10^−8^ and 2.19 × 10^−8^, respectively, *p* = 0.0395). In Figure 4B, the two adjuvants induced anti-P27A responses with similar EC_50_. Interestingly, increasing the dose of GLA-SE from 2.5 to 5 μg induced responses with higher functional affinity in the pre-exposed group (Figure 4C) in addition to higher titers. Therefore, in this population, affinity correlated with IgG titers (Figure 4D). This was not the case for unexposed subjects, for which data is not shown. Finally, adjuvant effect on affinity to 20-mer peptides P1, P5, P8, and P10 was analyzed using phase 1a samples that were positive for these peptides. Peptides showed low affinity compared to P27A LSP, with no difference between adjuvants (Figure S2).

We also measured the adjuvant effect on IgG avidity to bound P27A by ELISA using the chaotropic agent guanidinium hydrochloride. Representative serum samples from the unexposed population were evaluated. IgG avidity for plastic bound P27A was significantly higher in the CH-Alum/50 group than in the CH-GLA2.5/50 group (Figure 4E). Additionally, we used SPR to measure the binding kinetics of eight representative affinity purified sera samples with the highest anti-P27A titer from groups CH-Alum/50, CH-GLA2.5/50, and TZ-GLA2.5/50 at D84. Binding kinetics were clearly concentration-dependent for all the tested individuals (Figure S3). The avidities to bound P27A showed K_D_ values within the nanomolar range in the three groups, with a tendency for a lower avidity in those vaccinated with GLA-SE (Figure 4F). This aligns with the results obtained by ELISA using a chaotropic agent (Figure 4E).

## Discussion

P27A formulated with Alhydrogel and GLA-SE tested in phase 1a and 1b clinical trials conducted in Switzerland and Tanzania demonstrated promising T and B cell responses. The GLA-SE formulation, showed the most promise, with the observation that the intensity of response to identical vaccine formulations was lower for pre-exposed volunteers than for unexposed volunteers (15). In this follow-up report, we intended to characterize the fine T and B cell responses directed to the antigen, focusing on the potential impact of adjuvants and the exposure status of volunteers to *P. falciparum*.

Given the unstructured nature of P27A, the defined fine specificity of cellular and humoral response was easily assessed by using overlapping 20-mer peptides covering the entire P27A sequence as previously shown for MSP2 family specific and constant regions (20). As measured by ELISPOT, the T-cell response specific to the ten 20-mer peptides was not particularly elevated. However, P27A elicited a low but positive response in 12 of 16 volunteers. This was also evident in the concomitant B-cell response found in all volunteers, without qualitative difference between formulations.

Examining the antigen specificity with the 20-mer peptides indicates that the entire P27A sequence is a target of the humoral response in both cohorts, with no statistical difference between unexposed and pre-exposed volunteers except for peptides five and seven. It is not clear whether the difference observed is due to the difference in genetic background or to pre-exposure to malaria. Of note, the most recognized 20-mer peptides are peptide fragments 1, 5, 8, and 10, where 1 and 10 cover the N- and C- termini of P27A. The N- and C- termini are generally more flexible than the interior segment and may therefore have a higher probability of adapting favorably to different antibody binding sites.

Direct ELISA to plastic-bound antigens and the concomitant inhibition of the response by soluble P27A and its 20-mer peptides determine the antigen specificity and the apparent K_D_ of the interaction between the single antigens and antibodies. Competitive ELISA results show that the apparent K_D_ for the interaction between antigen and antibodies is around 10^−8^ M in vaccinated subjects. Nevertheless, pre-exposed volunteers exhibit significantly higher affinity, probably indicating a higher immune maturation due to the boost from the three injections of the vaccine in addition to the natural immunity induced by previous malaria exposure.

Moreover, increasing the dose of GLA-SE in the formulation favored higher anti-P27A titers (15), higher affinity and a reduction of the number of epitopes recognized. These are signs of an efficient humoral maturation, and corroborate the observation reported by Hill et al. (21) regarding the repertoire of Tfh and antibody secreting cells (ASC) of the volunteers from group TZ-GLA-SE5/50. Hill et al. observed that the high anti-P27A responses induced by GLA-SE are correlated with high oligoclonal expansion of extrafollicular ASC and Tfh expressing specific TCR (21).

Interestingly, the Alum formulation seems to induce an anti-P27A response with higher avidity than the GLA-SE formulation, particularly by ELISA. This discrepancy may be due to a technical bias. The avidity ELISA assay using a chaotropic agent measures the strength of Ig binding to plastic-bound peptides, while the affinity inhibition ELISA assay measures the strength of Ig binding to a soluble peptide in a native conformation. Because P27A is intrinsically unstructured and therefore flexible, its conformation is probably more prone to alteration when adsorbed to the plastic surface of the ELISA plate. The vaccine formulated in Alum, in which the P27A is absorbed on aluminum salt surfaces, vaccine may possibly induce responses to altered bound-peptides, at least partially. On the contrary, P27A in the GLA-SE emulsion will favor antibodies preferably or exclusively directed to and with high affinity to the soluble native P27A, so bound P27A is recognized with less affinity, and therefore with less avidity.

Moreover, while the adjuvants influence the magnitude of the immune response, studies have observed that they have less influence on the fine specificity and affinity of antigen-specific antibodies (22). The dose of adjuvant was making an exception to this, since we observed that a significant increase in affinity correlated with increased titers and the dose of GLA-SE in pre-exposed volunteers.

In conclusion, the two P27A vaccine formulations using two different adjuvants, Alhydrogel and GLA-SE, largely exhibit similar characteristics at the T and B cell level, independent of the intensity of the responses induced. Genetic differences between the Swiss and Tanzanian cohorts and previous exposure to the malaria parasite are likely to shape the subsequent immune response to antigen vaccination, which may explain observed differences in humoral responses between the two populations. The formulation of P27A in 5 μg GLA-SE is likely to induce an efficient immune response in pre-exposed populations, the target population that could benefit from the vaccine. This formulation may also be suitable for unexposed travelers since recent trials showed that 2 or 5 μg GLA-SE vaccine formulations were similarly safe (23, 24). Further studies to formally confirm these observations should be performed in phase 2a and 2b clinical trials covering a greater number of volunteers.

## Data Availability Statement

The datasets generated for this study are available on request to the corresponding author.

## Ethics Statement

The phase 1a study involving human participants was reviewed and approved by Commission cantonale d'éthique de la recherche sur l'être humain of the Canton de Vaud (CER-VD), Lausanne, Switzerland. The phase 1b study involving human participants was reviewed and approved by the National Institute For Medical Research (NIMR), Dar es Salam, Tanzania. The patients/participants provided their written informed consent to participate in these studies.

## Author Contributions

GC and RA were responsible for conceptualization. KG, DG, J-PB, and RA were responsible for investigation. CY and BC were responsible for SPR analyses. SH, RA, CM, CD, and FS were responsible for the clinical trial. KG was responsible for writing the original draft. RA, KG, GC, and FS were responsible for writing, reviewing and editing the manuscript.

### Conflict of Interest

The authors declare that the research was conducted in the absence of any commercial or financial relationships that could be construed as a potential conflict of interest.
