# Supplementary figures and images for: Epitope Mapping and Fine Specificity of Human T and B Cell Responses for Novel Candidate Blood-Stage Malaria Vaccine P27A
Source: Front Immunol. 2020 Mar 10;11:412. doi: 10.3389/fimmu.2020.00412 (PMC7076177; doi:10.3389/fimmu.2020.00412)

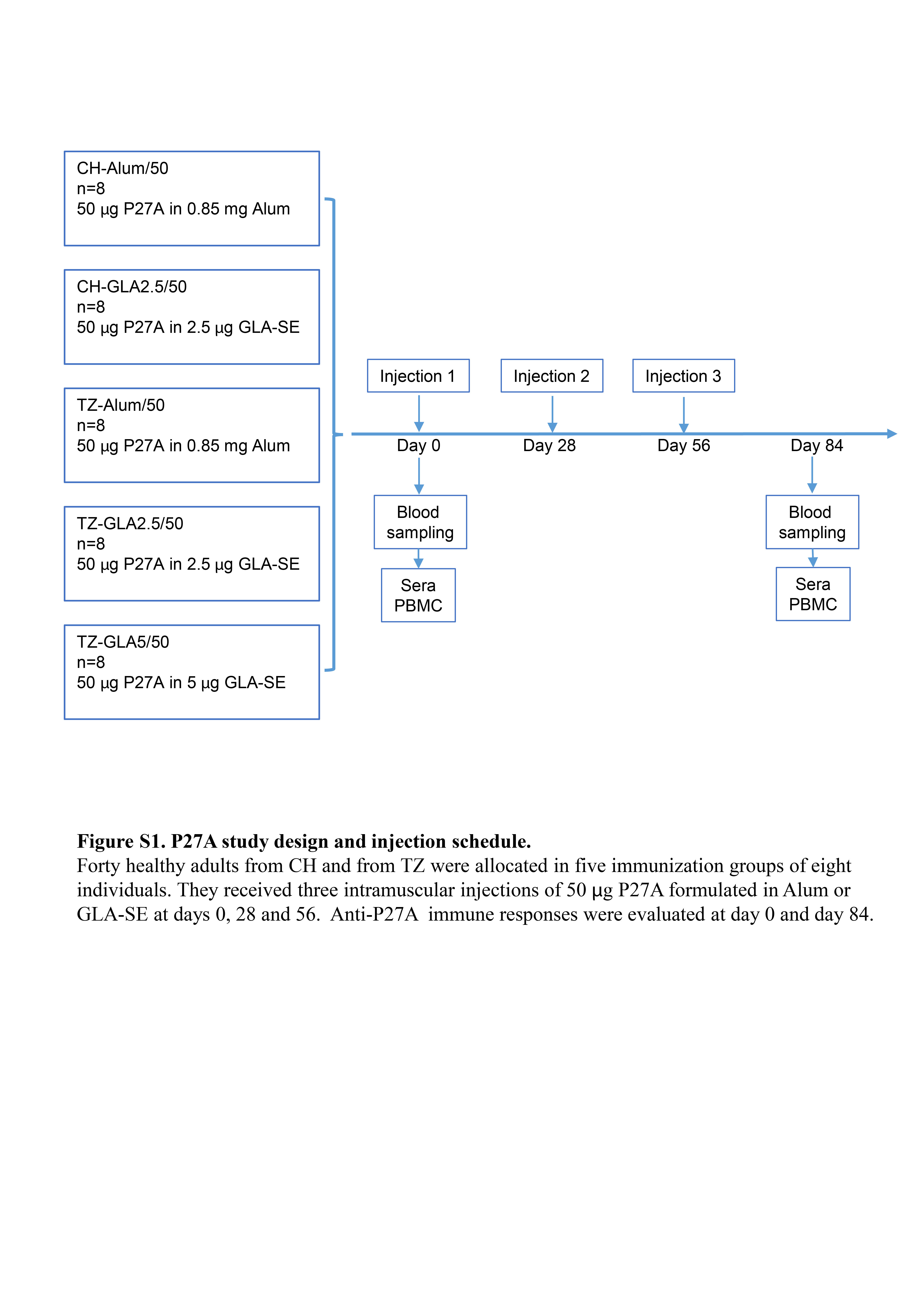

Supplement: Supplementary file 1 [file Image_1.tif]

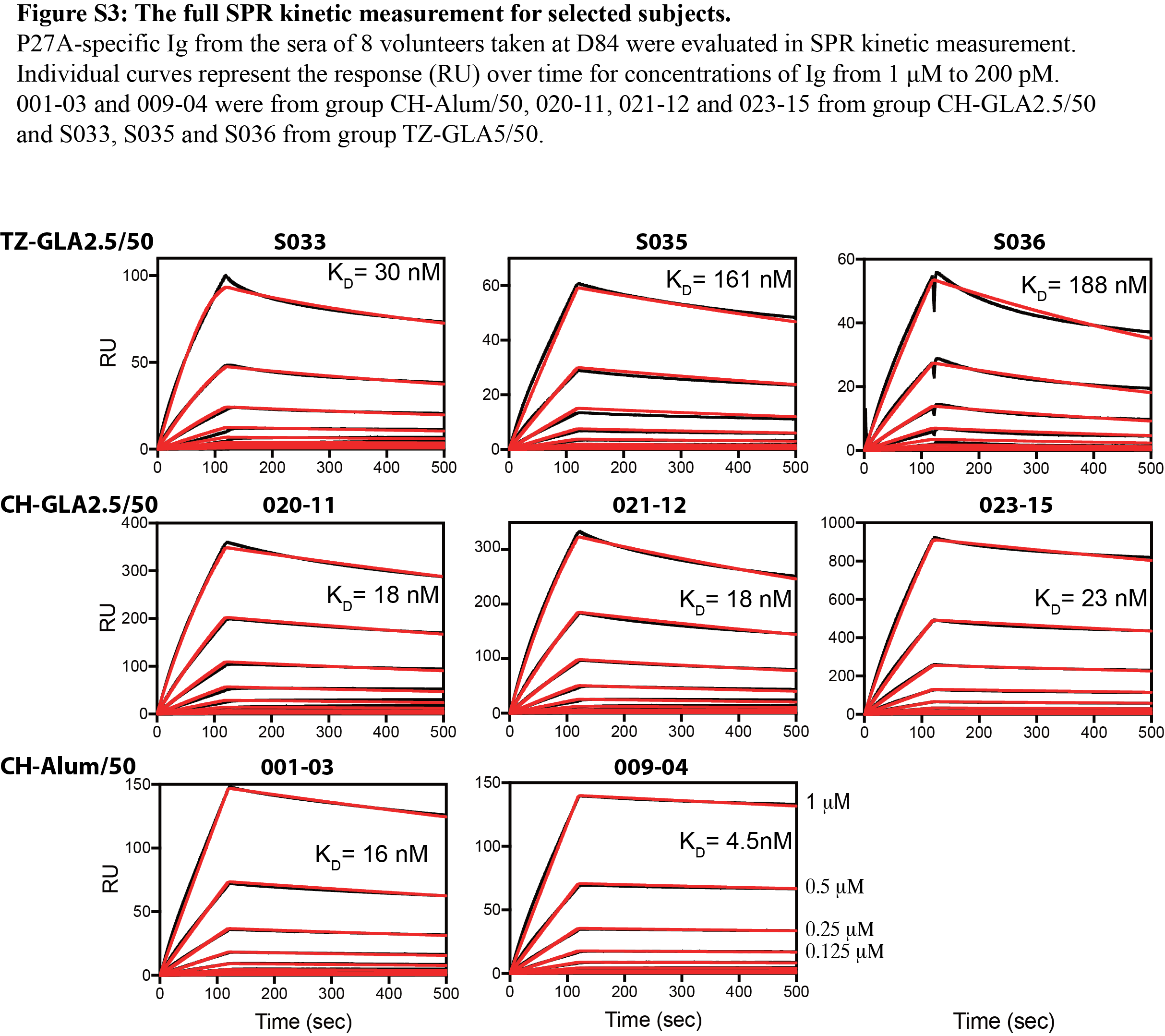

Supplement: Supplementary file 3 [file Image_3.TIF]
